# Design of Large-Scale Microwave Cavity for Uniform and Efficient Plastic Heating

**DOI:** 10.3390/polym14030541

**Published:** 2022-01-28

**Authors:** Sangjun Jeon, Jaekyung Kim, Daejong Yang

**Affiliations:** 1 Department of Future Convergence Engineering, Kongju National University, Cheonan 31080, Korea; A20200301@smail.kongju.ac.kr; 2 Industrial Technology Research Institute, Kongju National University, Cheonan 31080, Korea; kjk8431@gmail.com; 3 Department of Mechanical and Automotive Engineering, Kongju National University, Cheonan 31080, Korea

**Keywords:** microwave heating, cavity, reflector, double waveguide, uniform heating, high heating efficiency

## Abstract

To reduce carbon emissions during heating in the manufacturing processes, microwave technology has attracted significant attention. Microwaves have considerable advantages over traditional heating methods, including more rapid heating, lower thermal damage, and eco-friendly processes. To apply microwaves to the manufacturing process, uniform and efficient heating is required. We analyzed the effect of various design parameters for uniform and efficient heating by changing the cavity heights, application of the reflector, and number and positions of waveguides. We conducted a numerical simulation and verified the findings by experiments. The results showed that a slight change in the cavity height altered the electromagnetic field distribution and heating parameters, such as the coefficient of variance and power absorption efficiency. With reflectors installed, 66% of cases exhibited better comprehensive evaluation coefficient (CEC) with consideration of uniform heating and power absorption. The spherical reflector showed 81% of cases, better than those of the ordinary model without a reflector. Furthermore, when double waveguides were installed, the average coefficient of variance (COV) was improved by 22%, and power absorption efficiency was increased by 53% compared to the single waveguide case. When the power applied to the waveguides was doubled, the average COV values improved by 18%. This large-scale analysis will be helpful in applying microwaves to actual industrial sites.

## 1. Introduction

Fossil fuels represent approximately 80% of the total energy sources as of 2020 [1]. Besides energy, chemicals produced from fossil fuels are used daily. They are also employed in various fields, such as petroleum, construction, and electricity [2]. However, the processes of manufacturing chemical products and the use of fossil fuels produce emissions of large amounts of carbon dioxide (CO_2_) (33.5 Gt of CO_2_ annually), which is a greenhouse gas. The emission of CO_2_ into the atmosphere causes global warming by rapidly increasing the temperature of the earth. There have been active discussions among countries around the world to reduce the use of fossil fuels. To manage CO_2_ emissions from the use of fossil fuels, clean renewable energy sources, such as sunlight and wind power, are being used instead of conventional fossil fuels. Moreover, technologies that reduce CO_2_ emissions in the process of converting fossil fuels into different energy sources, such as electricity, are being employed. Although clean energy production and various efforts to reduce CO_2_ emissions are improving, the consumption of fossil-fuel-based plastic products is rapidly increasing each year, compromising the process of low-carbon transitioning. Therefore, there is a need to reduce the large amount of CO_2_ emissions from plastic-producing processes. To address this problem, many countries have implemented carbon reduction policies that mainly target carbon emission reduction during plastic production. Researchers have focused on various methods to reduce carbon emissions in various processes, such as sintering, drying, and synthesis. Among these processes, material processing using microwave heating has attracted significant attention [3].

Traditional heating methods of heat transfer involve heat radiation, convection, and conduction. These methods require several hours to reach the target temperature since the heat must be transferred from the heat source to the outside of the target material and then to the inside. This involves high energy consumption and long-term exposure to high temperatures, which leads to surface damage and property deterioration [4]. In contrast, in microwave heating, heating is performed by directly vibrating molecules or through particle charging [5,6]. Heating with microwaves requires less time than traditional heating [7]. Furthermore, it is eco-friendly because it consumes less energy and emits lower amounts of carbon than those in conventional heating. According to Gupta [8], carbon emissions can be reduced by several dozen times compared to the traditional heating process if the materials are heated using microwaves. Owing to this advantage, various studies have focused on heating using microwaves. For example, carbon nanotubes and hydrogen with higher qualities were produced by recycling plastics using microwaves, showing that this process was simpler than conventional methods [9]. Suriapparao [10] produced biodiesel fuel through microwave pyrolysis after mixing biomass with plastics. That author emphasized that the pyrolysis process that uses microwave heating is a method for obtaining fuel in an eco-friendly manner. In addition, research has been conducted on various processes using electromagnetic waves instead of traditional heating processes such as, nanomaterial synthesis, sintering, pyrolysis, and drying [11,12,13]. In particular, scholars reported that microwaves, a type of electromagnetic wave, are eco-friendly and more efficient than conventional heating methods [14,15,16].

Despite these advantages, microwave heating has the shortcoming of non-uniform heating. In traditional heating processes, the temperature difference between the surface and inside of the target material is a problem. However, in the microwave heating process hot spots with high temperature in specific areas, caused by the intrinsic characteristics of microwaves, degrade the quality of the target material and burn or explode the material in severe cases [17]. To address this problem, numerous studies have proposed several methods to improve the uniformity of heating [18]. For example, He applied rotary radiation to increase the heating efficiency and uniformity [19]. Ye applied a screw propeller to avoid thermal runaway and obtained a high heating uniformity [20]. Yi used a conveyor belt and a mode stirrer together and obtained a better heating performance than using them individually [21]. In addition, research has been conducted to improve heating performance by changing the geometry of the cavities [18,19,20,21,22,23,24]. Several studies applied multiple waveguides [23,24]. As another approach, Hong analyzed the heating performance by changing the frequency and power of the microwave and position of target materials [25]. Domínguez-Tortajada studied the heating behavior of dielectric layers for electric field uniformity [26].

Despite such studies, the application of microwaves in industries remains challenging for the following reasons. First, most experiments have been performed on a laboratory scale. In addition, only about 1/10 to 1/1000 of the total cavity volume is filled with the target materials, so only the heating of a local area in the center of the cavities is considered. This, this research cannot provide cavity design guides on a large-scale for industrial sites. Second, most studies are focused on heating efficiency, not uniformity. For example, Fu [27] studied the heating efficiency of plastic particles, not heating uniformity, by varying the microwave power and chamber volume. Specifically, studies on large-scale heating focus more on high efficiency. In several studies on drying or biodiesel production high heating efficiency is also the major interest [28,29]. However, focusing only on heating efficiency and not considering uniform heating can lead to a serious result. For example, overheating regions deteriorate the performance of the reactor and the quality of the material [17]. Thus, it is necessary to increase heating uniformity and energy efficiency simultaneously in industrial sites.

In this study, the hopper model, a large-capacity equipment model that is applicable to actual industrial sites, was designed. The results of a numerical analysis were compared with the experimental results. Various cavity heights, the application of the reflector, and the number and positions of waveguides were considered. The electromagnetic field distribution and heating effect according to the design parameters were calculated through the simulation to analyze the influence of each parameter. The results of this study can be applied to actual industrial sites and contribute to realizing a sustainable society.

## 2. Simulation and Experiments

### 2.1. Numerical Model

#### 2.1.1. Simulation Domain and Modules

**Figure 1:**
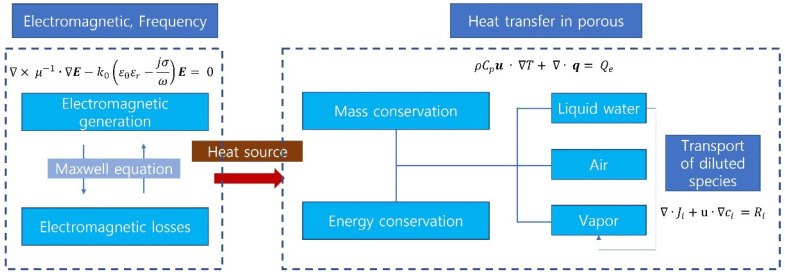
Simulation relationship between electromagnetic module and heat transfer in porous and transport diluted species module.

To process a material using microwave heating, it is essential to predict the temperature profile of the target material [23]. However, it is difficult to visually examine the temperature profile and electromagnetic field distribution inside a closed metal cavity during an actual test. Therefore, the temperature profile and electromagnetic field distribution were predicted through computer-based finite element analysis. Commercial finite element analysis software (COMSOL Multiphysics^®^ V5.6, COMSOL Inc., Stockholm, Sweden), which is widely used for microwave heating analysis, was used for the numerical simulation. The simulation was conducted under the frequency-transient condition using the heat transfer, electromagnetic, and transport diluted species modules. Figure 1 shows the relationship and interaction among the modules. The microwaves generated in the electromagnetic module form an electromagnetic field distribution inside the cavity based on Maxwell’s equations. The heat transfer module is associated with the electromagnetic module, and the electromagnetic field loss is regarded as a heat source in the heat transfer part. Based on the calculated heat source, the temperature and moisture distribution of the target material were derived from the heat transfer and transport diluted species modules. All these processes occurred according to the energy-mass conservation law.

#### 2.1.2. Geometry

**Figure 2:**
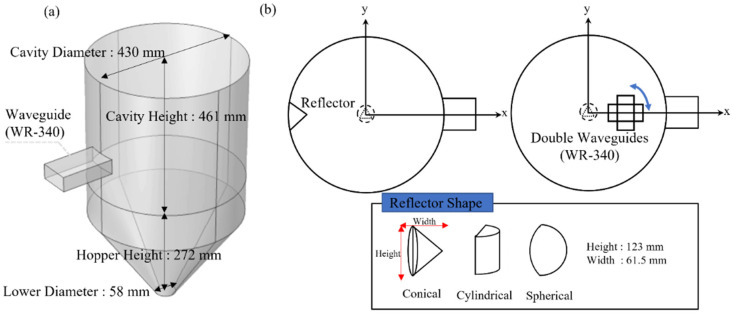
(**a**) Geometry of microwave hopper structure and (**b**) design parameter of simulation for microwave heating simulation.

Figure 2a shows the cavity model with the hopper structure used in the experiment and analysis. A 2.45 GHz magnetron (frequency spectrum from 2448 to 2458 MHz) was attached to the cavity side face, which operated in the transverse electric 10 mode (TE10). Microwaves with a power of 1 kW were generated by the magnetron and propagated into the cavity through rectangular waveguides. The attached waveguides had dimensions of WR-340 (a width of 86.4 mm and a height of 43.2 mm), and the simulation was performed with varying positions and several waveguides. The dimensions of the cavity and the design parameters to be applied in the simulation are summarized in Figure 2a,b. The cavity was cylindrical and had a diameter and height of 430 and 461 mm, respectively. The lower hopper had a height of 272 mm, an upper diameter of 430 mm, and a lower diameter of 58 mm. The height of the cavity was adjusted based on the wavelength of the 2.45 GHz microwave. The cavity walls and waveguides were made of copper, which reflects microwaves. In addition, 25 kg of polycarbonate was placed inside the hopper. Table 1 summarizes the material properties of the cavity and the target material required for analysis.

#### 2.1.3. Assumption

The following assumptions were applied to the analysis model to efficiently simulate complex phenomena during the microwave heating [30].

1. The cavity walls and waveguides are in the impedance condition;
2. The cavity walls and waveguides are completely insulated;
3. All materials are homogeneous;
4. Chemical reactions are not considered in the heating process;
5. The heat transfer model applies only to polycarbonates;
6. Shrinkage of pellets is not considered;
7. The influence of air flow is not considered;
8. The dielectric constant and loss factor are independent of temperature and humidity;
9. The frequency of the microwave is fixed at 2.45 GHz, which remains constant.

#### 2.1.4. Governing Equations

In this simulation, the governing equation of the electric field wave is given by the following equation [31]:

(1) $$\nabla \times \mu^{-1}\cdot \nabla E-k_{0}\left(\varepsilon_{0}\varepsilon_{r}-\frac{j\sigma }{\omega }\right)E=0$$

where E is the electric field vector, ω is angular frequency, σ is the conductivity, $\varepsilon_{0}\left(8.854\times 10^{-12}F/m\right)$ is permittivity of free space, and $k_{0}$ is the wave number of free space, which is defined as [19]

(2) $$k_{0}=\omega /c_{0}$$

where $c_{0}$ is the speed of light in vacuum.

The relative permeability of $\mu_{r}$ can be defined as [32]:

(3) $$\mu_{r}=\mu^{\prime }-\mu^{\prime \prime }$$

where $\mu^{\prime }$ is the magnetic constant, that is, a measure of the ability of the dielectric materials to store magnetic energy, $\mu^{\prime \prime }$ represents the loss of magnetic field energy.

The relative permittivity of $\varepsilon_{r}$ can be defined as [33]:

(4) $$\varepsilon_{r}=\varepsilon^{\prime }-j\varepsilon^{\prime \prime }\;$$

where $\varepsilon^{\prime }$ is the dielectric constant that represents the ability to store electromagnetic energy, and $\varepsilon^{\prime \prime }$ is the loss factor that is known to dissipate the absorbed electromagnetic energy, converting electromagnetic energy into heat.

When electromagnetic waves interact with the material, the electromagnetic energy is converted into heat based on Fourier’s energy balance equation within the area. The heat transfer in the hopper model can be expressed by Equation (5) [17].

(5) $$\rho C_{p}\frac{\partial T}{\partial t}+\rho C_{p}u\;\cdot \;\nabla T+\nabla \cdot \;q=Q_{e}$$

where ρ is the density, $C_{p}$ is the specific heat capacity, u is the velocity field from Darcy’s law, and T is the temperature. Heat flux q can be defined as [17]:

(6) q=−k∇T

where *k* is the thermal conductivity.

The electromagnetic loss, $Q_{e},$ is regarded as the heat source on the right side of Equation (7). $Q_{e}$ can be defined as [22]:

(7) $$Q_{e}=Q_{rh}+Q_{ml}$$

where the resistive losses are [34]:

(8) $$Q_{rh}=\frac{1}{2}Re\left(J\cdot E^{*}\right)$$

and the magnetic losses are [34]:

(9) $$Q_{ml}=\frac{1}{2}Re\left(i\omega B\cdot H^{*}\right)$$

J in Equation (8) is the current density. In Equation (9), B is the magnetic flux density, and H is the magnetic field intensity.

The existence of moisture plays an important role in heating. Water evaporation and heat convection can affect the temperature. When the temperature reaches a certain level, the phase and distribution of moisture will change, which can influence the heating effect; hence mass transfer processes should be considered. The mass transfer solved in polycarbonates is expressed as [4]

(10) $$\nabla \cdot J_{i}+u\cdot \nabla c_{i\;}=R_{i\;}$$

where $c_{i\;}$denotes the concentration of species, u is the fluid velocity, $R_{i\;}$is the molar production constant, and $J_{i}$ is the diffusive flux vector, which is given as [4]:

(11) $$J_{i}=-D_{i}\cdot \nabla c_{i\;}$$

where $D_{i}$ is the diffusion coefficient.

#### 2.1.5. Boundary Condition

##### Electromagnetic Boundary Conditions

Boundary conditions of impedance are applied on the cavity wall and waveguides where the electromagnetic field is known to penetrate only a short distance outside the boundary [34]:

(12) $$\sqrt{\frac{\mu_{0}\mu_{r}}{\varepsilon_{0}\varepsilon_{r}-j\sigma_{e}/\omega }}n\times H+E-\left(n\cdot E\right)=\left(n\cdot E_{s}\right)n-E_{s}$$

where $E_{s}$ is the source electric field and n is the normal vector toward the exterior of the target boundary.

In this study, the rectangular waveguides were excited by the frequency of the 2.45 GHz magnetron that operated in the TE10 mode.

The port boundary conditions are expressed as [22]:

(13) $$\beta =\frac{2\pi }{c_{0}}\sqrt{v^{2}-v_{c}^{2}}$$

where β is the propagation constant, v is the frequency of microwave, and $v_{c}$ is the cutoff frequency.

##### Thermal Condition

The thermal insulation boundary condition means that there is no heat flux, which can be expressed by the following equation [4]:

(14) −n·(−k∇T)=0

##### Mass Condition

Convection is applied to the target and can be expressed as [4]

(15) $$-n\cdot \left(-D\nabla c\right)=J_{0}$$

(16) $$J_{0}=k\left(c_{b}-c\right)$$

where *D* is the moisture diffusion coefficient in the sample, $c_{b}$ is the air moisture concentration, k refers to the mass transfer coefficient, and $J_{0}$ is the bottom face of the sample and is defined under the no-flux boundary condition.

#### 2.1.6. Mesh

**Figure 3:**
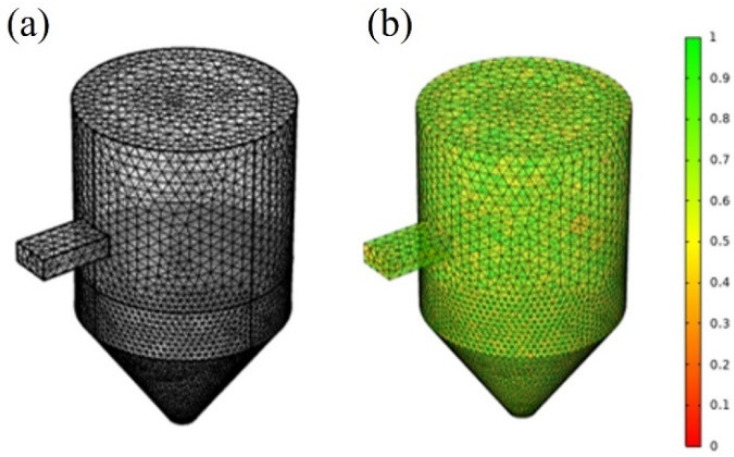
(**a**) Mesh element and (**b**) mesh quality evaluation using COMSOL Multiphysics^®^.

The mesh size is an important element in the simulation as it affects the convergence and accuracy of the analysis. For example, when the mesh size decreases by half, the computation time increases 16 times, and the memory usage increases eightfold [19]. According to the results of a previous study on numerical simulations, at least five mesh elements per wavelength are recommended for proper mesh size in microwave heating [35]. The mesh element quality (MEQ), one of the most commonly used methods, was evaluated by applying five or more mesh elements per wavelength for accurate simulation. In COMSOL Multiphysics, MEQ can be calculated using the following equation [36,37].

(17) $$\mathrm{MEQ}=\frac{4\sqrt{3}A}{h_{1}^{2}+h_{2}^{2}+h_{3}^{2}}$$

where A is the area and $h_{1}$, $h_{2}$, and $h_{3}$ are the side lengths of the triangle. 227,146 meshes were used. As shown in Figure 3, the average mesh quality was 0.9, which exceeded the criterion (0.65) of previous studies.

### 2.2. Experiments

#### 2.2.1. Experimental Setup

**Figure 4:**
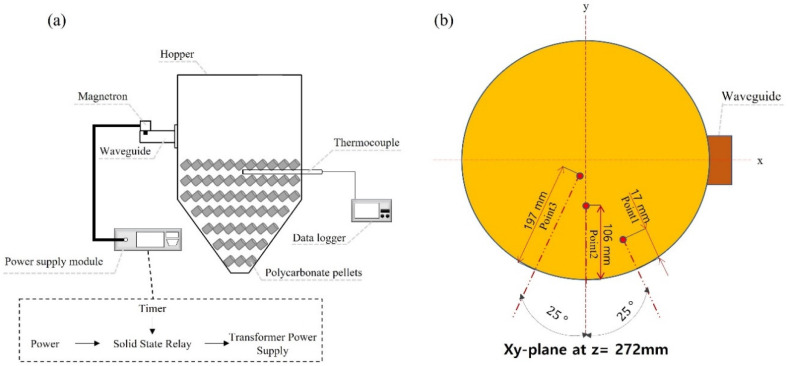
(**a**) Schematic of the experimental setup for microwave heating and (**b**) temperature sampling points.

Figure 4a shows the experimental setup, and its specifications are the same as those mentioned in the simulation. As shown in Figure 4b, only one 1 kW magnetron was attached to the 221 mm position on the side, and another magnetron was attached to the upper part at a position 120 mm away from the center. The densely filled polycarbonate pellets ensure reliable contact between thermocouples and target materials. The round-ended thermocouples were grounded to prevent spark.

#### 2.2.2. Experimental Procedure

Polycarbonate was exposed to a humid environment for several hours to create conditions similar to actual industrial environments. To prevent the difference in heating effect caused by the moisture content difference, the moisture content was maintained at 0.11% by monitoring it using a moisture meter (MS-70, AND, Tokyo, Japan). After inserting 25 kg of polycarbonate pellets into the experimental setup, the magnetron was operated for 600 s. The temperature of the polycarbonate was measured every 2 s using a thermocouple. The environment temperature was maintained 25 °C using Air-conditioning system.

#### 2.2.3. Experiment Validation

**Figure 5:**
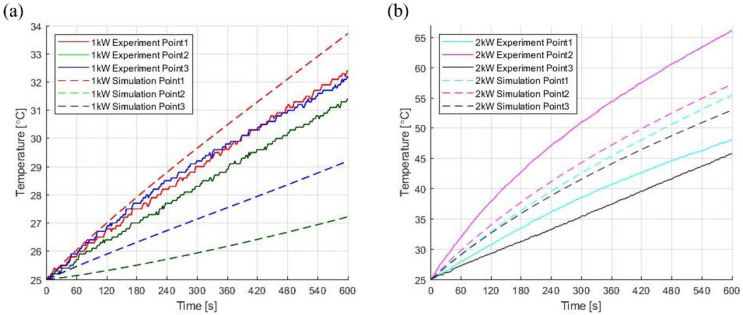
Temperature comparison between numerical simulation and experiment under (**a**) 1 kW and (**b**) 2 kW.

To verify the validity of the simulation model, the results of the experiment described above were compared with the simulation results. The atmospheric temperature at the time of the experiment, 25 °C, was set as the initial value, and the temperature values at the same positions as in the experimental setup were collected while 1 kW and 2 kW microwaves were radiated inside the cavity for 600 s. As shown in Figure 5, the root mean square error between the experimental values and simulation results were 0.791, 2.565 and 1.982 °C at the measuring depth of 17, 106 and 197 mm, respectively, when heating was performed using 1 kW for 10 min and 3.224, 6.408, 6.971 °C at the same points when 2 kW was supplied by installing two waveguides. The small errors between the simulation and experimental results confirmed that the numerical analysis model is reliable.

## 3. Results and Discussion

### 3.1. Parameters

Microwaves are generated from a magnetron and transmitted to the microwave cavity through the waveguide. In this instance, various design parameters, such as the cavity height, geometry of the reflector inside the cavity, positions and number of microwave port that reflect the microwaves in different directions, create various electromagnetic field distributions. The created electromagnetic field converts the energy of the microwaves into thermal energy through interactions with objects, thereby causing different heating results. As mentioned in the Introduction, reducing hot spots through uniform heating is an important element for microwave-based heating. The following coefficient of variance (COV) has been widely used to quantify the heating uniformity after microwave irradiation [38]:

(18) $$\mathrm{COV}=\frac{1}{E\left(T\right)}\sqrt{\frac{1}{N}\sum_{1}^{N}{\left(T_{i}-T_{0}\right)}^{2}}$$

(19) $$E\left(T\right)=T_{a}-T_{0}$$

where *T_i_* is the temperature at a specific point, *T_a_* is the average temperature of the target material to be heated, and *T*_0_ is the initial temperature. A lower COV value implies that the target material is heated more uniformly. The power absorption efficiency (PAE, η), which is a value indicating the energy absorption efficiency, has also been widely employed together with COV to understand the heating phenomenon [39].

(20) $$\eta =\frac{P_{a}}{P_{i}}$$

Here, *P_i_* is the total energy supplied to the model, and *P_a_* is the absorbed power, which is calculated through the volumetric integration of the power loss density. A higher PAE value indicates that the supplied energy is more efficiently converted into heat. In this study, the positions and sizes of hot spots that occurred inside the cavity were confirmed through an electromagnetic field distribution, and the heating uniformity and efficiency were analyzed for various parameters using the COV and PAE values.

### 3.2. Effect of Cavity Height

**Figure 6:**
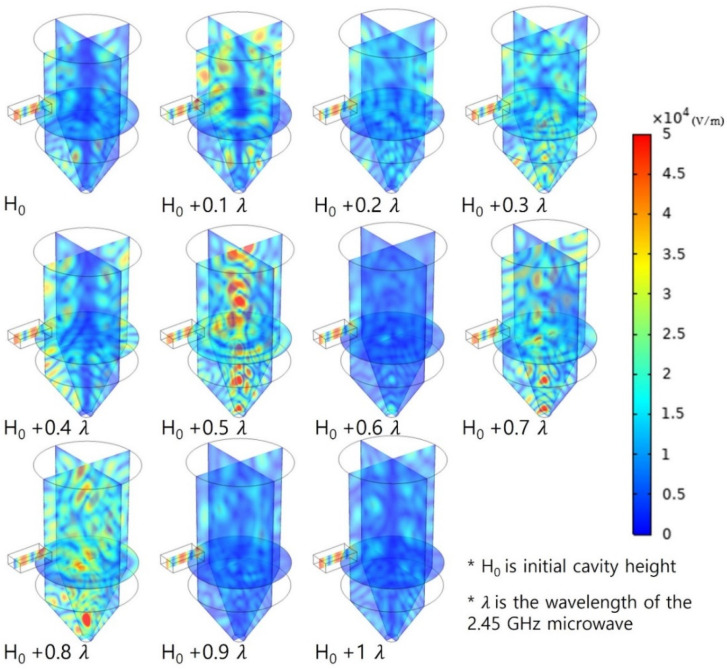
Electromagnetic field distribution by increasing the height of the cavity from 0 to 100% of the wavelength.

Since the distribution of the electromagnetic field changes considerably depending on the geometry of the cavity, the analysis was conducted according to the cavity height first. The cavity height was increased by 12.2 mm, which corresponded to a tenth of the wavelength of the 2.45 GHz microwave from 461 mm. The total increase was 122 mm. Figure 6 shows the electromagnetic field distribution according to height. Finding the pattern of the electromagnetic field distribution according to height using the electromagnetic field distribution map alone was challenging, as was predicting the distribution of hot spots and cold spots. For example, in the case of the cavity whose height was increased by 10% of the wavelength, cold spots were formed in the central part, whereas hot spots were formed in parts close to the surface. When the cavity height was increased by 50% of the wavelength, cold spots were formed toward the surface and wide hot spots were formed in the central part. This showed significant changes in the electromagnetic field despite a small change in geometry. Moreover, it showed that it is almost impossible to intuitively predict the electromagnetic field distribution.

**Figure 7:**
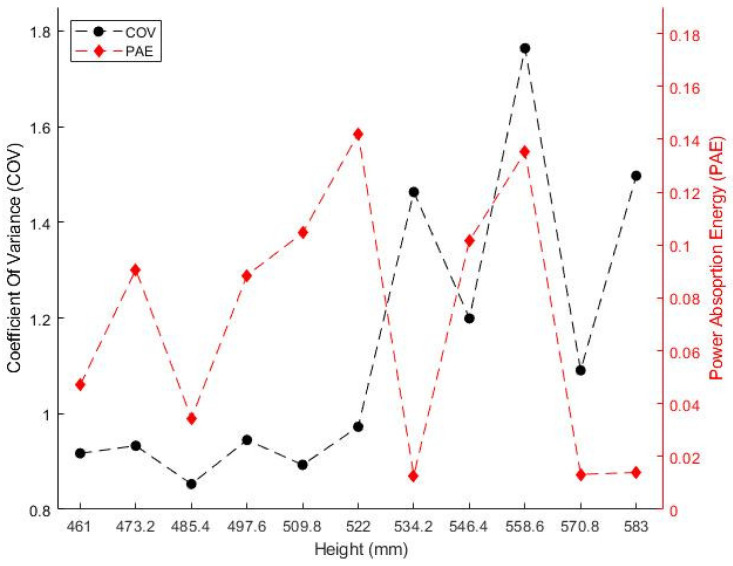
Coefficient of variance (COV) and power absorption efficiency (PAE) along cavity height.

To examine the heating effect according to height, the COV and PAE are summarized in Figure 7. When the height was increased by 20% of the wavelength, the largest COV value of 0.852 was observed, and the uniformity value became approximately two times higher than that when the height was increased by 80%, of the wavelength and the lowest COV value was observed. The heating efficiency also significantly changed depending on height, similar to the heating uniformity. When the height was increased by 50% of the wavelength, 141 W of overall power was absorbed. This occurred when the PAE value was 0.141 (14.1%), and the absorption rate was approximately 11 times higher than when the height was increased by 60% of the wavelength and the lowest PAE value was observed. A comparison between the PAE value and the electromagnetic field distribution summarized in Figure 6 reveals that the PAE value was also high when a strong electromagnetic field was generated. The positions and number of hot spots in the target material changed depending on the microwave cavity height owing to changes in the electromagnetic field distribution and the amount of electromagnetic energy passing through the polycarbonate. This resulted in differences in the temperature distribution and energy absorption efficiency. It indicated that the cavity height is a design parameter that significantly affects the heating effect and uniformity.

### 3.3. Effect of a Reflector

When an object is irradiated with microwaves, three reactions occur; that is, some waves are reflected, some pass through, and some are absorbed. Transparency and absorption are intrinsic characteristics of the material and vary depending on the wavelength of the irradiated microwaves. Reflection could be related to the target material, although it mainly occurs in the cavity because the reflectivity of the target material is relatively low. Thus, the heating pattern can be adjusted by changing the geometry of the cavity because it changes the energy distribution of microwaves. In this regard, research has been widely conducted to achieve more uniform heating than before the use of internal modification by adjusting the reflection of electromagnetic waves through a change in the cavity geometry or by installing objects, such as a rotating body or a conveyor belt, inside the cavity [40]. In this study, spherical, cylindrical, and conical reflectors that reflect microwaves were installed inside the microwave cavity, and changes in the electromagnetic field and the heating effect were analyzed. To evaluate the performance of the cavity in various directions, the comprehensive evaluation coefficient (CEC), which considers the heating efficiency and heating uniformity, as well as the PAE and COV, is defined as follows:

(21) CEC = PAE/COV

A higher CEC value indicates a higher energy absorption rate and more uniform heating. Therefore, CEC can be used as a comprehensive parameter [41].

#### Effect of Reflector Shape

**Figure 8:**
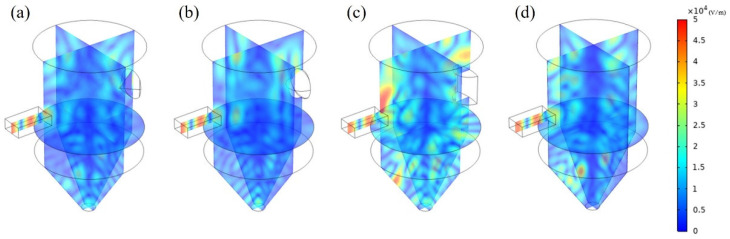
Electromagnetic field distribution with different reflector shapes such as (**a**) conical (**b**) spherical (**c**) cylindrical (**d**) no installation.

Conical, spherical, and cylindrical reflectors were placed inside the cavity and on the opposite side of the magnetron. Their dimensions and geometry are summarized in Figure 2. To analyze the influence of the installed reflectors on the internal environment and heating effect, a simulation was performed by applying three types of reflectors and changing their cavity heights. As can be observed from the electromagnetic field distribution in Figure 8, more notable changes in the electromagnetic field were observed when the reflectors were installed than when only the height of the cavity was changed. The highest intensity of the electromagnetic field with no height change was 4.82 × 10^4^ V/m for the existing model; however, it increased by approximately 60% to 7.84 × 10^4^ V/m when a conical reflector was installed. The spherical and cylindrical reflectors also increased the highest intensity of the electromagnetic field to 4.88 × 10^4^ and 5.82 × 10^4^ V/m, respectively.

The simulation was performed for 44 cases while varying the reflector geometry (4 cases) and cavity height (11 cases); the COV and PAE values are summarized in Table 2. Whether more uniform heating occurs in the presence of reflectors was analyzed based on the case with no reflector. Among the 33 cases with reflectors, lower COV values were observed in the presence of reflectors in 19 cases (57%), confirming more uniform heating than in the models with no reflector. In addition, the PAE value was higher in the presence of reflectors in 21 cases (63%) out of the 33 cases, showing that the installation of reflectors can increase the energy absorption. In particular, for the cavity whose height increased by 20% of the wavelength compared to the ordinary model, the energy absorption was approximately 3% when no reflector was installed, while it increased at least twofold when any of the reflectors was installed.

**Figure 9:**
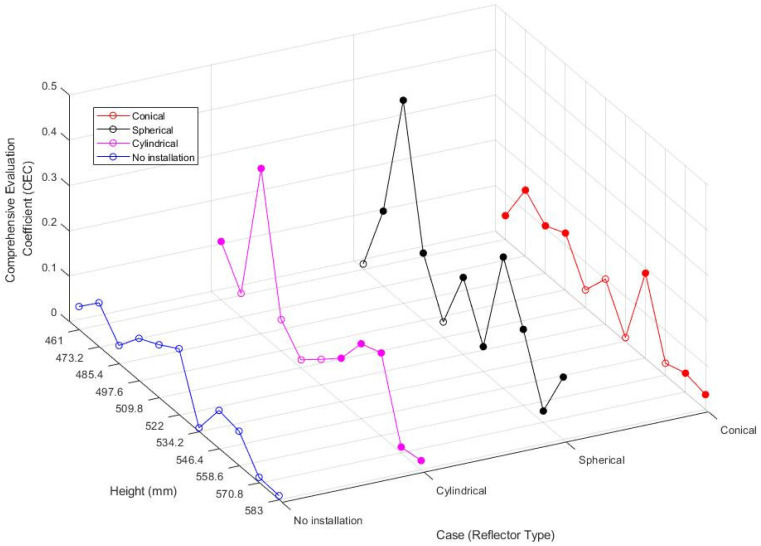
Comprehensive evaluation coefficient (CEC) with different reflector shapes and heights of the cavity.

Figure 9 shows the CEC values for 44 cases. For the CEC value that reflected both the energy efficiency and heating uniformity, 66% (filled marks in Figure 9) exhibited better values than the ordinary model with no reflector. When the reflectors were compared, the spherical and conical reflectors exhibited better performance than the cylindrical reflector. When only the spherical and conical reflectors were considered, 16 cases out of 22 (72%) exhibited higher CEC values than the model with no reflectors. In the case of the spherical reflector, 9 cases out of the 11 (81%) showed better performance. This appears to be because the spherical and conical reflectors reflected electromagnetic waves in three dimensions, whereas the cylindrical reflector reflected them in two dimensions. The installation of reflectors was found to be an important factor for cavity design as it changed the electromagnetic field distribution more notably even though it did not always lead to more uniform and higher heating efficiency.

### 3.4. Effect of Double Waveguides

In the preceding chapters, changes in the electromagnetic field and heat distribution due to changes in cavity geometry were analyzed. Changing the cavity geometry and the method of using multiple waveguides have also been proposed as measures to achieve uniform and high-efficiency heating [23]. However, most studies have focused on the application of multiple waveguides to the fixed cavity. However, studies on the influence of detailed cavity design parameters, such as the position, port direction, and power, on the electromagnetic field distribution and heating effect are insufficient and remain on a laboratory scale. Therefore, more waveguides were installed in the cavity of various geometries, and the temperature uniformity and energy efficiency of the target material during heating were analyzed.

#### 3.4.1. Effect of Double Waveguides, Position, and Direction

**Figure 10:**
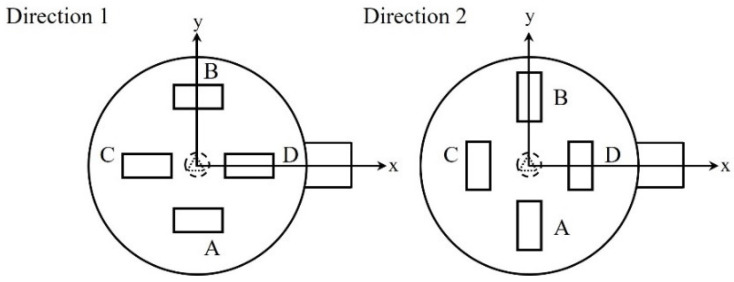
Schematic illustration presenting the position of the additional waveguide.

To analyze the influence of the double waveguides, another waveguide was installed in the upper part in addition to the existing waveguide position. The waveguides were vertically arranged on the side and in the upper part of the cavity to reduce the resonance and offset between the electromagnetic waves from them. Figure 10 summarizes the geometry and installation positions of the waveguides. In the case of the waveguide placed in the upper part, eight cases were simulated based on four positions and two directions. The two waveguides had dimensions of WR-340, and each waveguide had a power of 0.5 kW. Accordingly, a total of 1 kW of energy could be emitted as in the single waveguide model.

**Figure 11:**
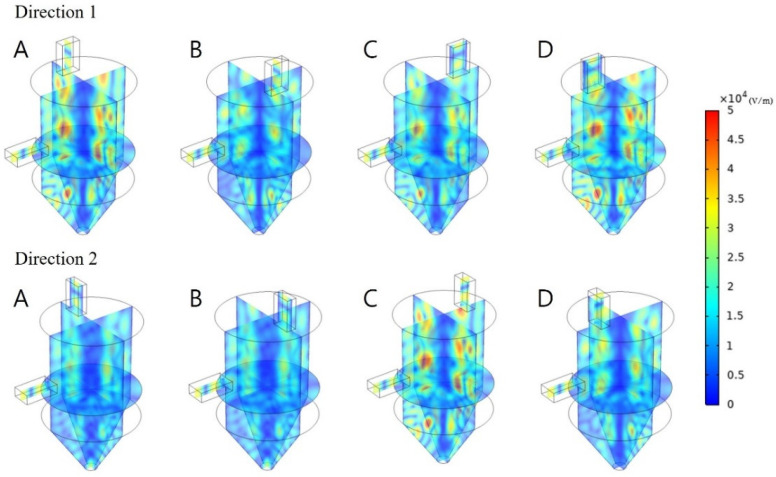
Electromagnetic field distribution with different reflector positions and directions under two 0.5 kW radiation sources.

As shown in the surface electromagnetic field distribution in Figure 11, a hot spot occurred at the top center of the target material when double waveguides were installed, and it was wider compared with the existing single waveguide model. When double waveguides were installed, the intensity of the electromagnetic field was found to be higher than the maximum intensity of the electromagnetic field in the existing model (2.54 × 10^4^ V/m), irrespective of the parameters.

Table 3 shows the COV and PAE for the eight positions and directions of the waveguides. First, in the case of Direction 1, the most uniform heating was observed when the waveguide was located at position D in Figure 11, where the COV value was found to be 0.659. All four positions were more uniformly heated than when the single waveguide was used, and the average COV value was 0.710, exhibiting 22% higher uniformity than when one waveguide port was used. The highest energy absorption was also observed at position D, similar to the COV, with the PAE value being 0.116. The PAE value was also higher at all positions than when the single waveguide was used. The average value of the four positions was 0.08, which was 22% higher than that of the ordinary model.

In the case of Direction 2, when the waveguide was located at position D, the most uniform heating was achieved among the eight cases, as the COV value was 0.566. The average COV value for the four positions was 0.701, which was higher than that when the single waveguide was installed. The average PAE value for the four positions was also 0.059, which was excellent compared to the case when the single waveguide was installed.

Consequently, when double waveguides were used, more excellent heating uniformity and energy absorption were observed in all eight cases. The use of the double waveguides made it possible to reach areas that were difficult to reach when microwaves were radiated from the side; by radiating microwaves in the vertical direction as well, more uniform heating occurred. The target material was uniformly heated, and the energy absorption increased compared to when the single waveguide was used because the cases of generating hot spots due to the concentration of electromagnetic waves caused by interference and cold spots due to the offset between the microwaves decreased.

#### 3.4.2. Effect of Double Waveguide Power

**Figure 12:**
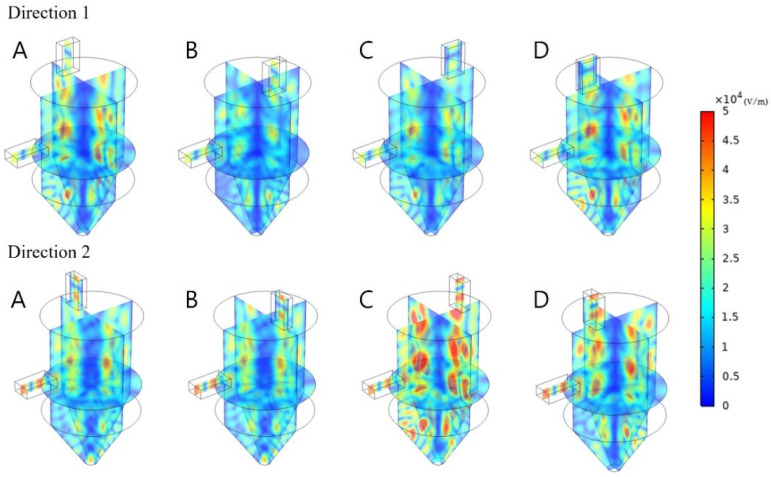
Electromagnetic field distribution with different reflector positions and directions under two 1 kW radiation sources.

In the preceding section, the effect of double waveguides was confirmed. For their application in industries, the heating effect according to supplied power is also an important element. Therefore, in this section, the heating effect was compared and analyzed while the power per waveguide was increased from 0.5 to 1 kW. The electromagnetic field distribution map in Figure 12 shows that when the power of the waveguides was increased, the distribution of the electromagnetic field remained similar, whereas its intensity increased. Thus, the position of the hot spot also remained similar, although its size increased.

When the COV and PAE values described in Table 4 were calculated, the COV value was found to decrease regardless of the position and direction, as the power per port increased from 0.5 to 1 kW. In particular, when the power was 1 kW in Direction 1, the average COV value for the four positions was 0.647, which was 117% lower than the case when the power was 0.5 kW. The energy efficiency remained similar as the input power doubled even though the energy absorption almost doubled.

Since the inside of the cavity was insulated, the average temperature also doubled as the energy supply doubled. As the positions of the waveguides remained unchanged, the temperature profile was maintained and only its intensity increased. The increased average temperature led to a relatively uniform temperature distribution because the absorbed heat could not be transferred to the outside of the cavity. This indicated that the boundary conditions, as well as the cavity geometry, are important design parameters for the cavity. The excellent insulation effect of the cavity can save energy and create a uniform temperature distribution by preventing the loss of thermal energy.

## 4. Conclusions

In this study, the microwave heating effect was analyzed on a pilot-scale according to the design parameters, such as the cavity height, installation and type of reflectors, and number and position of waveguides. The numerical analysis results were verified by comparison with the experimental temperature profile, and the following conclusions were drawn:

(1) Despite a slight change in the cavity height caused by changing the wavelength by one-tenth, the electromagnetic field distribution and parameters were considerably changed. The COV value, which means heating uniformity, and the PAE value, which means heating efficiency, were changed up to double and 11 times, respectively;
(2) The installation of reflectors significantly changed the electromagnetic field distribution. When reflectors were installed, uniform heating was generally achieved, and the power absorption also increased. The spherical reflector had the highest efficiency; 9 out of 11 cases showed better CEC values with a comprehensive coefficient that reflects both PAE and COV.
(3) When double waveguides were used, the target material was uniformly heated, and the energy absorption increased at the same time because electromagnetic waves reached areas that were difficult to reach using the single waveguide. So, the average COV was improved by 22%, and power absorption efficiency was increased by 53% compared to the single waveguide model. These results came from the reduction of interference phenomenon by the vertical arrangement of waveguides.
(4) As the applied power increased, the form of the electromagnetic field distribution did not change, although the size of the hot spot increased. The average COV value was 0.647, which was 117% lower than the basic model.

The heat inside the cavity increased owing to the increased power supply, and the increase in the average temperature of the target material led to uniform heating. The above observations revealed that the cavity geometry, application of reflectors, arrangement of waveguides, and insulation conditions of the cavity are important parameters for cavity design. This study provides the way microwave heating applies to actual industrial sites. Based on the uniform and efficient heating shown in this study, considering the various factors such as chemical reactions, phase change, etc., in the future, it is expected to be applicable to processes such as drying, synthesis and decomposition which are widely used in industry.

## Figures and Tables

**Table 1:** Physical properties of polycarbonate.

No.	Parameters	Value or Expression	Reference
1	Microwave frequency	2.45 GHz	-
2	Microwave power	1 kW	-
3	Air moisture concentration	$0.05\;\mathrm{mol}/m^{3}$	[4]
4	Relative permittivity of polycarbonate	$2.35-j2\times 10^{-4}$	[9]
5	Thermal conductivity ^1^	$0.07182401\;W/\left(m\cdot K\right)+7.942708\times 10^{-4}\;W/\left(m\cdot K^{2}\right)\cdot T$ $-1.8525\times 10^{-6}\;W/\left(m\cdot K^{3}\right)\cdot T^{2}+2.126779\times 10^{-9}\;W/\left(m\cdot K^{4}\right)\cdot T^{3}$	-
6	Polycarbonate density ^1^	$1050\;\mathrm{kg}/m^{3}$	-

^1^ is from the COMSOL built-in material library.

**Table 2:** COV and PAE values with different reflector shapes and heights of the cavity.

Height (mm)	Evaluation	Conical	Spherical	Cylindrical	No Installation
461	COV	0.638	1.349	0.612	0.917
	PAE	0.033	0.016	0.079	0.047
473.2	COV	1.861	2.010	1.138	0.933
	PAE	0.271	0.348	0.058	0.091
485.4	COV	0.646	0.856	0.640	0.853
	PAE	0.067	0.384	0.233	0.034
497.6	COV	0.690	0.979	1.095	0.945
	PAE	0.087	0.145	0.074	0.089
509.8	COV	1.549	1.773	1.240	0.893
	PAE	0.059	0.060	0.021	0.105
522	COV	0.982	0.795	0.898	0.973
	PAE	0.098	0.135	0.050	0.142
534.2	COV	1.607	0.800	1.183	1.463
	PAE	0.013	0.043	0.113	0.013
546.4	COV	1.090	0.582	0.683	1.199
	PAE	0.205	0.168	0.113	0.102
558.6	COV	1.575	0.978	0.894	1.764
	PAE	0.042	0.164	0.163	0.135
570.8	COV	0.800	0.738	1.218	1.091
	PAE	0.034	0.019	0.015	0.013
583	COV	1.244	0.679	1.650	1.497
	PAE	0.0401	0.093	0.033	0.014

**Table 3:** Coefficient of variance (COV) and power absorption efficiency (PAE) for different reflector positions and directions under two 0.5 kW radiation sources.

Direction	Evaluation	A	B	C	D	Average	Single Waveguide
1	COV	0.686	0.780	0.717	0.659	0.710	0.917
	PAE	0.081	0.052	0.080	0.116	0.082	0.046
2	COV	0.830	0.831	0.578	0.566	0.701	0.917
	PAE	0.050	0.050	0.087	0.049	0.059	0.046

**Table 4:** Coefficient of variance (COV) and power absorption efficiency (PAE) of different reflector positions and directions under two 1 kW radiation sources.

Direction	Evaluation	A	B	C	D	Average
1	COV	0.631	0.666	0.663	0.628	0.647
	PAE	0.081	0.052	0.080	0.116	0.082
2	COV	0.827	0.829	0.576	0.565	0.699
	PAE	0.050	0.050	0.087	0.049	0.059

## Data Availability

Data available in a publicly accessible repository.
